# The Rate of Antibiotic Dosage Adjustment in Renal Dysfunction

**Published:** 2012

**Authors:** Fanak Fahimi, Sepideh Emami, Farin Rashid Farokhi

**Affiliations:** a *Clinical Pharmacy Department, School of Pharmacy, Shahid Beheshti University of Medical Sciences, Tehran, Iran.*; b *Chronic Respiratory Disease Research Center (CRDRC), NRITLD, Masih Daneshvari Hospital, Shahid Beheshti University of Medical Sciences, Tehran, Iran.*; c *Telemedicine Research Center, NRITLD, Masih Daneshvari hospital, Shahid Beheshti University of Medical Sciences, Tehran, Iran.*; d *Department of Nephrology, Masih Daneshvari hospital, Shahid Beheshti University of Medical Sciences, Tehran, Iran.*

**Keywords:** Renal dysfunction, Dosage adjustment, Creatinine clearance, Cockcroft and Gault, MDRD.

## Abstract

The purpose of this study was to determine the number of prescribed antibiotics being appropriately adjusted and to assess antibiotics with the highest incorrect dosing based on the patient’s renal function according to distinguished guidelines.

The study was conducted at a 446-bed university hospital. One hundred and fifty patients admitted through different wards of the hospital were included in the study. Demographic data were extracted and creatinine clearance was calculated using either Cockcroft-Gault (C&G) or Modification of Diet in Renal Disease (MDRD) formula. In patients with creatinine clearances less than 50 mL/min, antibiotic dosages were compared with guideline dose recommendations to judge whether they were correctly adjusted.

Two hundreds and ninety-one instructions (79.9%) of 364 antibiotic prescriptions required dosage adjustment based on the patient’s renal condition. These adjustments were rationally performed in 43.7% and 61.4% of prescriptions, according to the two guidelines used. Ciprofloxacin (29.1% of cases), and vancomycin (33.6% of cases), were the most inappropriate prescribed antibiotics in terms of dose administration.

Drug dosing adjustments should be emphasized in patients with renal dysfunction. Failure to do so may lead to higher morbidity and mortality as well as therapeutic costs. Estimating creatinine clearance prior to drug ordering and use of a reliable dosing guideline is highly recommended.

## Introduction

The accumulation and toxicity of many drugs can develop rapidly if dosages are not adjusted in patients with renal impairment (1). Kidney disease studies have shown that the cases of chronic kidney disease are growing dramatically over the past 20 years (2, 3).

More than half of the adverse drug effects are due to the inappropriate dosage adjustments (4). Correctly adjusting the drug dosage in renal dysfunction, contributes to fewer adverse drug effects and decreases the therapeutic costs, (5) hospitalization, length of hospital stay and mortality as well as maintaining therapeutic effectiveness (6).

Even though not common yet, different strategies such as setting automated alerts and dosing checks for physicians’ prescribing drugs to patients with renal failure had been conducted, where the latter did not seem to be improved enough to comply with available dosing guidelines especially in the elderly and patients with unstable renal function (4).

Our analysis was focused on the following objectives: 1) determining the percentage of antibiotics requiring dosage adjustment in patients with kidney disease according to the available guidelines; 2) assessing antibiotics with the most inappropriate prescribed dosages based on the patient’s renal condition.

## Experimental

*Setting*

The study was performed from April 2008 to July 2009 at a teaching hospital; a 446-bed center specialized for pulmonary diseases located in northern Tehran, Iran. The hospital consisted of internal, surgical, oncology wards and intensive care unit.

*Design*

The medication records of patients older than 16, who were admitted to the hospital, were reviewed. The charts with at least one antibiotic needing dosage adjustment in kidney disease were included at the first stage. Since most drugs need dosage adjustment when creatinine clearance falls below 50 mL/min, (4) patients whose creatinine clearances fell below 50 mL/min were assigned for later analysis. Demographic data including sex, age and weight were also extracted. The creatinine clearance was calculated using either Cockcroft and Gault (C & G) (7), or the Modification of Diet in Renal Disease (MDRD) equation (8). The beds in our center were not equipped with scale. The Cockcroft and Gault formula was used in patients with accurate accessed weight, whereas for patients in intensive-care-unit (ICU) as well as disabled ones, the MDRD equation was chosen to estimate Glomerular Filtration Rate (GFR) due to the fact that no weight record was available. The prescribed dosages were then compared with available guideline dose recommendations (Guideline 1 (G1): AphA’s Drug Information Handbook (9) and Guideline 2 (G2): Drug Prescribing in Renal Failure: Dosing Guidelines for Adults (10), to assess whether they are appropriately adjusted according to the patient’s renal function. Since either weight or drug concentration is needed for the precise dosing calculations for amikacin, patients on this drug with no weight record were also excluded from the study (G1,2). Furthermore, imipenem doses could not be adjusted precisely in patients with no weight record based on “G2”.

Data was analyzed using SPSS version 16.0 (SPSS, Inc., Chicago, IL). Descriptive statistics (frequency, percentage, mean, standard deviation) were used to examine the normality of the data and describe the analysis.

## Results and Discussion

A total of 150 patients (57 females and 93 males) with a mean ± SD age of 61.6 ± 17.1 years were recruited.

Totally, 364 antibiotics were ordered. Eighty-seven and 47 patients were in internal wards and ICU, respectively.

Patients’ mean serum creatinine ± SD were 2.5 ± 1.9 mg/dL. Renal function indices are summarized in Table 1. 

**Table 1:** Patients’ demographics

**Patient data**	**Mean ± SD (Range)**
Male, n (%)	93(62%)
Age (yr)	61.6 ± 17.1 (21-87)
SCr^a^ (mg/dL)	2.5 ± 1.9 (1.1-16.4)
CrCl^b^ (mL/min)	34.4 ± 11.0 (10.5-49.9)
30-50, n (%)	93 (62.0%)
10-30, n (%)	46 (30.6%)
< 10, n (%)	11 (7.3%)

^a^SCr = Serum Creatinine Concentration; ^b^CrCl = Creatinine Clearance

After the exclusion of patients with no weight record for the named medications, of 364 antibiotic prescriptions, 281 and 285 of the prescribed antibiotics required dosage adjustment according to patient’s renal function consistent with G1 and G2, respectively. Among the antibiotics needing dosage adjustment, 43.7% and 38.5% were adjusted appropriately based on G1 and G2, respectively. Ciprofloxacin (PO/IV) was the most frequently prescribed antibiotic requiring dosage adjustment (37.5%) followed by vancomycin (22.0%). Ciprofloxacin (PO/IV) was the antibiotic with the highest (29.1% of cases) unadjusted dose using AphA’s handbook as the reference (Table 2). In most cases of inappropriate dosage adjustment (90%), the guideline’s recommended dosing intervals were not followed. Of the unadjusted doses, 143/86 incorrect interval cases (G1/ G2), 14/23 (G1/G2) incorrect dose, and in one case (G1 and G2) incorrect dose and interval were observed.

About 61.4% of the prescribed dosages complied with the second guideline’s dose recommendations (10), where vancomycin was the most inappropriately prescribed antibiotic (33.6%) (Table 2). Furthermore, in 33 patients (22.0%), the adjustments were performed for all antibiotics requiring dosage adjustment. For 59 patients (39.3%), adjustments were not performed in any of the antibiotics requiring dose adjustment.

**Table 2:** The rate of prescribed antibiotics which needed dose-adjustment using two guidelines

**Antibiotic**	**Prescribed (n)**	**Not Adjusted, n (%)**	
		**Guideline 1 (G1)** ^a^ **(n = 158)**	**Guideline 2 (G2)** ^b^ **(n = 110)**
Ciprofloxacin (PO/IV)	45/64	46 (29.1)	15 (13.6)
Vancomycin (IV)	64	44 (27.8)	37 (33.6)
Ceftazidime (IV)	39	28 (17.7)	27 (24.5)
Piperacillin (IV)	24	12 (7.5)	10 (9)
Cefixime (PO)	16	11 (6.9)	11 (10)
Meropenem (IV)	13	8 (5)	7 (6.3)
Imipenem (IV)	9	3 (1.8)	-----
Cefuroxime (IV)	2	2 (1.2)	-----
TMP/SMX ^c^ (PO)	2	2 (1.2)	2 (1.8)
Amoxicillin / Clavulanate Potassium (PO)	2	1 (0.6)	-----
Cefazolin (IV)	3	1 (0.6)	1 (0.9)

There was no correlation between the rate of inappropriate dosing and the calculated GFRs.

A total of 291 antibiotic prescriptions (79.9%) required dosage adjustment according to the patient’s renal function. Dosage adjustments were appropriately implemented in nearly 43% and 60% of these prescriptions consistent with two guidelines used, respectively. Other studies have also focused on inappropriate drug dosage adjustment in renal failure. In one study (4), it was observed that 1/3 of patients have different degrees of kidney disease at discharge, in 23.9% of which, the drug dosage adjustments were required according to the patient’s renal function and these adjustments were performed in only 58.9% of the cases, which is in accordance with our result, despite the fact that the setting and the reference were different for adjustment.

Another study (11) was performed on drugs with high fractional renal clearance among the inpatients with estimated creatinine clearance ≤ 40 mL/min. The prescribed dose within 30% of recommended dosage considered to be appropriate. Results of the latter study indicated that drug dosages were high in 44.8% of prescriptions requiring dose adjustment according to the patient’s renal condition. The cut-off point for creatinine clearance used in this study may explain the difference in the findings. A profound variation exists among the results of different studies on drug dosage adjustment. These variations are mainly due to the different equations chosen to predict GFR and also the variety in guideline’s recommendations on drug dosage adjustment. The simplified, easily applied MDRD equation (which was used to estimate GFR for a considerable number of patients in our study), does not take the patient’s weight or height into account and as a result, may fail to make an accurate prediction of GFR.(8). The above mentioned formula cannot be completely relied on, especially while prescribing drugs to patients with unstable renal function, since their GFR is already unpredictable due to the rapid and abrupt changes in serum creatinine levels. Although the methods based on timely urine collection can be performed to predict GFR in the mentioned group of patients, (12) either of equations developed from different studies to estimate creatinine clearance, should not be applied in patients with rapidly changing serum creatinine values, since these equations are derived from studies on individuals with normal or stable renal function (7, 8). Therefore, we recognized the above mentioned problem as a limitation of our study.

Furthermore, it had been validated that common sources of drug information differ in their definition and recommendations for drug dosage adjustments and dosing intervals in renal dysfunction (13). These remarkable variations make it difficult to decide which guideline dose recommendation is more reliable. As a result, these recommendations should not be applied in a clinical setting without involving the clinician or pharmacotherapist interpretation.

The results also suggest that not all clinicians estimate creatinine clearance prior to prescribing drugs to patients with renal dysfunction. In a study aimed to asses resident’s prescribing behavior in renal failure, it was revealed that only a few residents (5%), requested drug dosage checking for patients with elevated serum creatinine and that 35% of the questioned physicians performed dosage adjustments only for serum creatinine levels above 1.7 mg/dL (14).

Attention should especially be drawn to patients at risk of developing renal dysfunction. Estimating renal function by calculating creatinine clearance rather than using serum creatinine values alone, helps recognize these patients and adjust drug orders accordingly. Moreover, an intervention might be necessary for studies focusing on dosage adjustments in renal failure. By performing these interventions in this patient population, the impact of correctly applied dosage adjustment on reducing the length of Hospital Stay and mortality can be assessed and improved as well, although this was not the objective of our study. In all steps of the aforementioned interventions, the role of clinical pharmacist is particularly important, leading to the correction of possible dosing errors in addition to optimizing pharmaceutical care for patients with renal impairment (4).

Subsequently, in patients receiving renal eliminated drugs, serum drug levels could be measured to correlate with observed adverse reactions. This correlation would indicate that whether these reactions are of significant clinical importance. Implementing computerized drug dosage checking alerts at the time of ordering, can also be helpful to decrease the inappropriate drug dosing, however further studies are still necessary to assess the impact of these alert systems on clinician’s drug dosing behavior (15).

## Conclusion

Approximately 80% of the studied antibiotic prescriptions required dosage adjustment based on patient’s renal function. These adjustments were rationally performed in 43.7% and 61.4% of prescriptions consistent with the two guidelines used, respectively. In another study conducted by the same author (FF) a few years ago on enoxaparin in the same institution, it was shown that the appropriate dosage adjustment was performed for only 4 out of 8 patients (50%) with creatinine clearance (ClCr) < 30 mL/min (16). The results demonstrate a significant need to develop a unanimous drug dosing system for patients with renal dysfunction. Since lack of uniformity exists among dosing recommendations of commonly used drug information handbooks, finding a reliable and easily applied dosing guideline is highly recommended. The results of our study may not be extrapolated to other clinical settings, since they represent a unique situation. Therefore, further research is still required to reveal the clinical importance of drug dosage adjustment in renal failure patients.
